# Mixed‐methods study protocol for explanation of pregnant women’s concerns about antenatal anomaly screening process: Designing, implementing and evaluation of intervention

**DOI:** 10.1002/nop2.1012

**Published:** 2021-07-30

**Authors:** Zohreh Khakbazan, Hamid Poursharifi, Farnaz Farnam, Sedigheh Hantoushzadeh, Parsa Abdollahi, Shima Haghani, Mitra Arjmandifar

**Affiliations:** ^1^ Department of Reproductive Health School of Nursing and Midwifery Tehran University of Medical Sciences Tehran Iran; ^2^ Department of Clinical Psychology University of Social Welfare and Rehabilitation Sciences Tehran Iran; ^3^ School of Medicine Vali‐e‐Asr Research Center Tehran University of Medical Sciences Tehran Iran; ^4^ School of Medicine Tehran Medical Sciences Islamic Azad University Tehran Iran; ^5^ Nursing care research center School of Nursing and Midwifery Tehran University of Medical Sciences Tehran Iran

**Keywords:** antenatal anomaly screening test, concerns, intervention program, mixed‐methods study, pregnant women

## Abstract

**Aim:**

This study aims to investigate the concerns of Iranian pregnant women in the antenatal anomaly screening process (AASP) and propose an intervention to reduce these concerns.

**Design:**

This exploratory sequential mixed‐methods study is conducted in three stages (qualitative, intervention design and quantitative), in Tehran.

**Methods:**

A qualitative study is carried out to collect pregnant women's concerns during the AASP. Then, a two‐step procedure is implemented. In the first step (expert session), the concerns extracted in the qualitative part are prioritized. Next, the interventions used to reduce the concerns of pregnant women in the AASP are reviewed by considering the priority determined in the previous stage. The information obtained from this step is used to design intervention. Ultimately, a randomized controlled trial is used to evaluate the effectiveness of the intervention.

**Discussion:**

The results can be used for framing policies in health systems to address pregnant women's concerns in the AASP and to promote their mental health.

**Abbreviations**
AASP: antenatal anomaly screening process
AASTs: antenatal anomaly screening tests
DASS: Depression, Anxiety and Stress Scale
NGT: nominal group technique
SPSS: Statistical Package for the Social Sciences
WK: weeks

## 1 INTRODUCTION

Chromosomal disorders with a prevalence of 4 in 1,000 live births are among the important determinants of paediatric health and a healthy population. Trisomies constitute almost half of all chromosomal abnormalities (Wellesley et al., 2012). Down syndrome is the most common non‐lethal trisomy and occurs one per 740 live births in the United States (Parker et al., 2010), indicating an increase of approximately 33% compared with the late 1970s (Shin et al., 2009). This increase in prevalence is associated with increased maternal age in this period. According to the guidelines of the American College of Obstetrics and Gynecology, all pregnant women should be offered aneuploidy screening or diagnostic testing in early pregnancy (ACOG & May, 2016).

According to the current birth rate in Iran, about 3,000 cases of Down syndrome are expected annually among all live births. As a result of growth in late marriage and pregnancy and increased maternal demand for foetal health screening, the relevant technological facilities available in Iran's health system should be used for Down syndrome screening. According to the Iranian National Guideline for prevention of foetal chromosomal abnormalities, Down syndrome and trisomy 18 and 13, screening tests are recommended to all pregnant women, and the purpose of the screening should be explained to them from Day 1. The current policy for antenatal anomaly screening tests (AASTs) in Iran, depending on the time of attending perinatal care centres, includes ‘the first trimester combined test’ (nuchal translucency, pregnancy‐associated plasma protein‐A and human chorionic gonadotropin, between 11 weeks (WK) and 13 WK and 6 days of gestation) or ‘quadruple tests’ (human chorionic gonadotropin, alpha‐fetoprotein, unconjugated estriol and inhibin‐A, between 14WK and 16 WK and 6 days of gestation) (Ministry of Health and Medical Education, 2017). In Iran, performing AASTs is voluntary but, due to the high costs of caring for children with Down syndrome, most people choose to have these tests performed despite their high costs. However, most pregnant women are covered by government insurance that pays most of the nuchal translucency and other foetal examination ultrasounds costs.

Regardless of the stress and anxiety associated with pregnancy‐related tests and ultrasound examinations, pregnancy alone can cause stress and anxiety in many women (Georgsson Öhman et al., 2004). Pregnant women might experience varying degrees of anxiety during pregnancy. In this regard, many women experience emotional distress during the first and third trimesters (Georgsson Öhman, 2005). It is very important to address maternal concerns during pregnancy because the mental state of pregnant mothers has a relationship with the occurrence of low birth weight and cesarean delivery (Bastani et al., 2006; van Bussel et al., 2009; Matthey et al., 2003; Robertson et al., 2004). These anxieties and worries affect the mothers' sleep quality, as well as the quality of life, and can have such psychological consequences as postpartum depression (Jomeen, 2004).

## 2 BACKGROUND

Little is known about the adverse effects of AASP on pregnant women and their families. Few studies have used qualitative approaches to explore mothers' concerns about AASTs. The majority of studies on women's concerns about AASTs have been conducted in developed countries (Allison et al., 2011; Kaasen et al., 2010; Leiva Portocarrero et al., 2017; Lou et al., 2016), whereas these concerns are expected to be different in developing countries.

Qualitative studies have showed women's concerns in the process of undergoing AASTs from different angles (Carroll et al., 2000; Chiang et al., 2006; Gitsels‐van der Wal et al., 2015; Gottfreðsdóttir, 2009; Lewis et al., 2016; Potter et al., 2008; Reid et al., 2009; Tischler et al., 2011; Williams et al., 2005). For instance, a systematic review, conducted by Reid et al., examined factors affecting pregnant women's decisions regarding accepting or rejecting AASTs. They identified five important themes (destination unknown; to choose or not to choose; the risk is rarely pure and never simple; treading on dreams and betwixt and between) (Reid et al., 2009). In another qualitative study, carried out by Lou et al. in Denmark, the experiences of women and their spouses were investigated while waiting for the results of diagnostic tests, as well as the strategies they adopted to cope with anxiety and distress. Based on the results of this study, all couples experienced anxiety and fear while waiting for the results of diagnostic tests and used strategies, such as isolation or social engagement, to deal with anxiety. However, no measure was taken to manage the waiting time in these cases (Lou et al., 2016). Other studies have explained the views, perceptions and experiences of general practitioners and midwives regarding counseling for AASTs (Dodampahala & Wijeratne, 2014; Gitsels–van der Wal et al., 2014; Martin et al., 2014; Nagle et al., 2008). For instance, Dodampahala qualitatively investigated how physicians and midwives provided counselling to obtain the consent of pregnant women for screening tests and concluded that the counselling process was insufficient and inadequate time was devoted to this issue (Dodampahala & Wijeratne, 2014).

As diagnostic results are normal in the majority of the women with high‐risk AASTs, these unreasonable concerns are the main psychological and social costs these women pay for screening tests. Promoting professional support during these stages might manage these concerns and their associated distress (Peñacoba‐Puente et al., 2011). In Iran, most of the studies on pregnancy concerns have a quantitative design (Kordi et al., 2015; Yousefi, 2015). Quantitative research in understanding the context in which people live is weak (Creswell & Plano Clark, 2018). Maternal concerns during pregnancy have cultural, social, economic and medical dimensions. Therefore, the first step in understanding this concept is to conduct qualitative studies. Furthermore, knowledge of maternal concerns during pregnancy makes it possible to adopt proper strategies to improve their health, quality of life and pregnancy experiences (Peñacoba‐Puente et al., 2011; Puente et al., 2011). There is not any proper qualitative study on the concerns of pregnant women during the AASP, especially on the existing socio‐cultural context of Iran.

The mixed‐methods studies are often based on the pragmatism philosophical approach. Instead of methodology, pragmatic mixed methods are concentrated on the primary research question(s). Although Peirce, Dewey and James are all known as pragmatists, their pragmatism fits more to quantitatively driven mixed methods, convergent or equal‐status mixed methods, and qualitatively driven mixed methods, respectively. In James' pragmatism, the focus is on the practical concepts and outcomes of certain approaches used to describe a phenomenon. Pluralistic ontological and epistemological studies are also emphasized in James' pragmatism (Younas et al., 2019). This study is based on James' philosophy of pragmatism. Based on this approach, the mixed use of qualitative and quantitative methods leads to a better insight into the studied phenomenon and provides much evidence for studying a research problem than either quantitative or qualitative research alone (Creswell and Plano Clark, 2018). According to this fact that there is little information and no specific interventions in the field of pregnant women's concerns about AASTs, the researcher intends to conduct a multiphase exploratory sequential study for explaining the pregnant women's concerns in the process of undergoing AASTs and to design and evaluate interventions in this regard.

## 3 PURPOSE OF THE STUDY

This exploratory sequential mixed‐methods study will be implemented in three stages (qualitative stage, intervention design stage and quantitative stage). The objectives of each stage are as follows:

### 3.1 Objective of the first stage: qualitative study

Explaining the pregnant women's concerns in the process of antenatal anomaly screening tests.

### 3.2 Objective of the second stage: intervention design

Designing an intervention program based on data extracted from the qualitative stage and literature review.

### 3.3 Objective of the third stage: quantitative study

Investigating the effect of designing intervention programs on reducing pregnant women's concerns in the process of antenatal anomaly screening tests.

## 4 METHODS

### 4.1 Study design

**FIGURE. 1:**
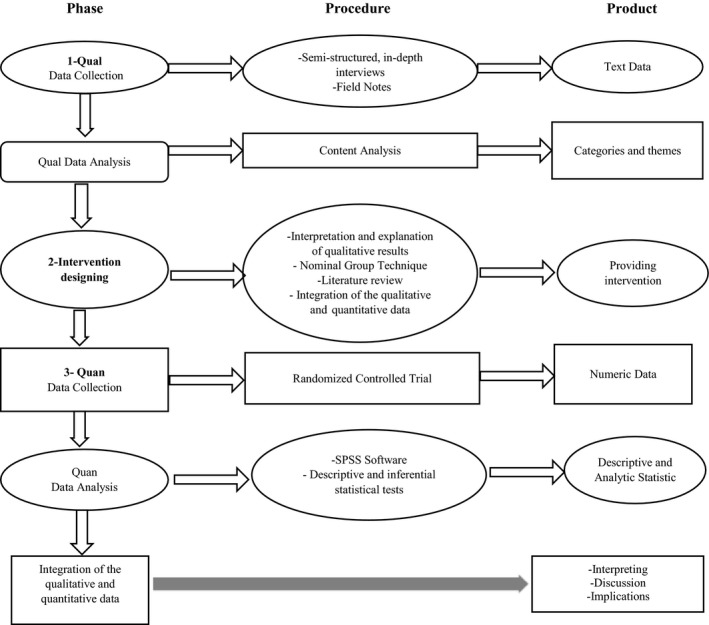
Study visual diagram

This is an exploratory sequential mixed‐methods study. An exploratory sequential design is a biphasic mixed study in which the researcher qualitatively explores the intended subject before building the quantitative study (Creswell and Plano Clark, 2018). In the first part of this study, a qualitative study with a content analysis approach will be conducted to explain the pregnant women's concerns associated with the AASP. To identify the concerns of pregnant women, individual, semi‐structured and in‐depth interviews will be conducted with a focus on pregnant women who have received counseling for undergone AASTs. Purposive sampling will continue in health centres and hospitals, affiliated with the Tehran University of Medical Sciences until data saturation is achieved. Afterwards, during the intervention design stage, a two‐step process will be carried out; in the first step, a panel of experts will be held, including the research team and a group of experts in various health fields with experiences in providing services to pregnant women. In this meeting, the concerns extracted in the qualitative part of the study will be prioritized using the Nominal Group Technique (NGT), and the panel's viewpoints will be collected. In the second step (designing the intervention), the interventions, programs and instructions to reduce pregnant women's concerns in the process of AASTs in Iran and other countries will be investigated by considering the main priority identified in the previous phase. The information obtained from this phase will be used to develop and design an intervention to reduce pregnant women's concerns in the AASP. Finally, in the final step (quantitative part of the study – intervention test), a randomized controlled trial will be performed. In the quantitative part of the study, after completing the questionnaires, data analysis will be performed using SPSS and descriptive and inferential tests. Finally, based on quantitative results, the researcher will interpret the extent to which the designed intervention has been able to reduce pregnant women's concerns about AASTs (Figure 1).

### 4.2 First stage: a qualitative study

The first stage of this study is designed to answer the question of ‘What are the concerns of pregnant women about antenatal anomaly screening tests?’ This study will be carried out using a qualitative content analysis method.

#### 4.2.1 Participants in the qualitative stage

The study population of the first stage will include pregnant women attending health centres and hospitals affiliated with Tehran University of Medical Sciences for prenatal care.

#### 4.2.2 Sampling method

In this study, the participants will be selected with a maximum variation in age, parity, the desirability of the pregnancy, job, education and social status, using purposive sampling.

#### 4.2.3 Inclusion criteria for participants

- Pregnant women attending perinatal care centres
- Iranian nationality and the ability to speak and understand Farsi
- Willingness to participate in the study and sharing relevant experiences

#### 4.2.4 Research environment

The participants are accessed through health centres and university hospitals affiliated with Tehran University of Medical Sciences, midwifery departments and perinatology departments. The interviews will be conducted at the participants' preferred time and place to ensure their comfort.

#### 4.2.5 Data collection process

Data will be collected after obtaining the approval of the Research Ethics Committee and receiving a letter of introduction from the Tehran University of Medical Sciences. In the first stage, in‐depth personal interviews will be used to collect data. These interviews will be based on an interview guideline. Before the interviews, informed consent will be obtained from the participants. Interviews will be conducted in a location comfortable for participants and will start with communication to win their trust. The participants will be then asked to express their views on their concerns about the AASTs. Subsequent questions will be asked based on the participants' initial answers and the interview guide. Besides, questions, such as ‘what do you mean or explain more please’, will be asked if needed. At the end of the interview, the researcher will ask the participants to state any other opinions and then will talk to them about the possibility of further interviews. The number of interview sessions depends on the participants and their answers to the research questions. The interviews will be recorded and transcribed verbatim with the participants' permission and will be presented to them to check their accuracy. As the interviews continue, the interview guide may be modified, and new questions will be added if necessary.

#### 4.2.6 Data analysis

The conventional content analysis approach will be used to analyse qualitative data. In this study, qualitative content analysis will be performed according to Graneheim and Lundman's method (Graneheim & Lundman, 2004). In this method, after each interview, the content will be transcribed at the earliest convenience. The statements will be read word by word several times to gain a general understanding of them. The semantic units will then be identified from the content of each interview. The semantic units – groups of words or phrases that have the same content – will be coded. Then, primary codes that centre around a central concept will be placed in a similar subcategory. Afterwards, the subcategories will be reviewed several times and checked for similarities and differences. Consequently, categories and themes will be formed. Attempts will be made to have the highest homogeneity in the categories and find the most heterogeneity between the categories. A computer‐assisted program MAXQDA 10 will be used to manage the data (Kuckartz, 2010).

#### 4.2.7 Determining the validity and reliability of data

Five criteria are suggested for reliability and validity analysis: credibility, dependability, transferability, confirmability and authenticity (Polit & Beck, 2008). Several methods will be used to confirm the validity: member check; external check; peer debriefing; prolonged engagement with the data; data triangulation, such as using interviews and field notes; and maximum variation sampling in terms of age, education, career, living place and stage of tests In this research, external check and peer debriefing will be used to confirm dependability, and samples of scripts and codes will be sent to the participants to monitor the data analysis process and apply their positive and critical opinions.

Due to the nature of qualitative research that lacks transferability, the researcher will try to accurately record the research path and the decisions made in this process and write her dissertation on the subject to enable other investigators to have a correct estimate of the generalizability of the findings. To this end, the researcher will accurately describe the participants, sampling method and time and place of data collection and will perform sampling with maximum variation in terms of age, living area, level of education, occupation and different social classes to enable the reader to use these findings in other contexts and situations to enhance the generalizability of the findings.

To ensure confirmability, interview transcripts, and extracted codes and categories will be provided to the research team and a faculty member to verify the accuracy of the process. Furthermore, different phases of the study will be presented very clearly to enable other participants to follow and judge the appropriateness of the research process To establish sincerity, the researcher will try to relate the participants' statements, feelings and experiences honestly. In addition, the audio files, interview transcripts and extracted codes and categories will be provided to the research team members to check the authenticity of the process and the reports.

### 4.3 Second stage: Intervention designing

Results from the data collection process in the qualitative stage will inform the data collection approach of the second stage procedure. In fact, the information obtained from the qualitative study will show us the search path in the studies and the selection of the intervention type based on the experts' opinions. Then the suitable intervention program will be selected and implemented in the quantitative phase based on this prioritization. In the second stage of the study, two steps will be completed for designing and implementing interventions to reduce the concerns of pregnant women.

#### 4.3.1 Prioritizing concerns

At this stage, the pregnant women's concerns, extracted in the qualitative part of the study, will be prioritized by investigating the views and opinions of the expert panel who have experience in providing health care or medical services to pregnant women. During this stage, an expert meeting will be organized using the NGT. This method supports the equivalent participation of group members as it limits the power of individual decision‐making and allows all members of the group to express their opinions equally by balancing each individual's influence (Wortley et al., 2016). This technique will be implemented in four stages, including generating ideas, reporting and recording ideas, discussing ideas and ultimately voting to rank ideas (Control and C. F. D. & Prevention., 2006). For voting, group members will be requested to select five of the most important recorded concerns (themes and categories), privately and anonymously, without consulting others and rank them according to their importance and feasibility of intervention design from 1 (least important) to 5 (most important). Finally, the researcher will count and combine the votes in the presence of all participants. Accordingly, a concern obtaining the highest score will be considered the most desired and important concern for which intervention will be designed.

#### 4.3.2 Designing the intervention

At this stage, the interventions and programs for promoting pregnant women's physical and mental health in Iran and other countries will be investigated considering the main priority set in the previous stage. Accordingly, Google Scholar, PubMed, ScienceDirect, Cochrane Library, Scopus, Magiran and SID will be searched, and different combinations of keywords will be reviewed between 2000 and 2020. Then, the related interventions and programs will be evaluated. Therefore, using the results of the qualitative study and reviewing the literature, a list of recommendations will be developed and prioritized. According to the information obtained from this stage, the target group, purposes and primary content of the intervention will be designed with the guidance of supervisors and advisors.

### 4.4 Joint displays

A joint display is a table that appears as a structure to discuss the integrated analysis and help both researchers and readers in understanding how a mixed‐methods provides new insights (Guetterman et al., 2015). We will have two joint displays in this study. A decision‐making matrix (first joint display) will be used for the prioritization of the extracted data from the qualitative study. This matrix consists of columns of ‘the themes and categories’, ‘scoring with respect to importance and the possibility to design interventions for themes and categories by the experts’, ‘the information obtained from the search process’ and ‘the type of the designed intervention’. We will use the second joint display to present a graphical and content analysis of the inferences from the qualitative and quantitative phases, and the comprehensive conclusions will determine by merging the individual inferences (Creswell and Plano Clark, 2018).

### 4.5 Third stage: A quantitative study

The quantitative stage of the study will be carried out using a randomized controlled trial.

#### 4.5.1 Research population

The research population of the quantitative study section includes all pregnant women presenting to the prenatal clinics of hospitals and health centres affiliated with Tehran University of Medical Sciences.

#### 4.5.2 Research sample

In this section, participants include pregnant women that meet the inclusion criteria and are presented to the prenatal clinics of hospitals and health centres affiliated with Tehran University of Medical Sciences.

#### 4.5.3 Research environment

This study will be carried out in health centres and prenatal clinics of hospitals affiliated with Tehran University of Medical Sciences.

#### 4.5.4 Sample size

The sample size will be 75 in each group with a confidence interval of 95%, trial power of 80%, Cohen's d of 0.5 and a sample loss of 15%.

#### 4.5.5 Sampling method

In the quantitative part of the study, convenience and continuous sampling will be implemented, in that all eligible individuals will enter the study after receiving a detailed explanation about the purpose of the study and signing an informed consent form. Afterwards, the samples will be divided into intervention and comparison groups using permuted block randomization based on Random Allocation Software considering all possible ways of assigning groups to blocks. The size of the blocks will be randomly selected (e.g., blocks containing 4, 8, 12 and 16 persons equally divided in each group). By selecting blocks randomly, the possibility of disclosing the last allocation in each block will be eliminated (Elkins, 2013; Kang et al., 2008).

This study will be an open‐label trial as masking of intervention is not possible. One of the researchers' colleagues will make the allocation sequence, and the researcher will register the participants and assign them to the intervention and control groups. All participating women will complete the initial questionnaires after randomization. The intervention group will be contacted and informed about the implementation of the intervention.

#### 4.5.6 Inclusion criteria

- Pregnant women with a gestational age of fewer than 11 weeks that apply for completing the initial prenatal record
- Lack of Down syndrome and other anomalies in the foetus in previous pregnancies
- No history of known mental disorders or consumption of psychiatric drugs
- Pregnant women with a significant level of worry about AASP

#### 4.5.7 Exclusion criteria

- Adverse incidents during the study
- Spontaneous abortion or death of the foetus during the study
- Lack of motivation to cooperate with the researcher

Inclusion and exclusion criteria in the quantitative part of the study will be modified and completed after implementing the qualitative part and determining the preferred intervention.

#### 4.5.8 Research variables

In this clinical trial, the designed intervention is considered an independent variable. Pregnant women's concerns in the process of performing AASTs are considered dependent variables. The dependent variables in the quantitative part of the study will be modified and completed after implementing the qualitative part and determining the preferred intervention.

#### 4.5.9 Data collection methods

Data from the quantitative study section will be collected using three questionnaires: the Depression, Anxiety and Stress Scale (DASS‐21), a researcher‐made questionnaire for measuring pregnant women's concerns in the process of AASTs, and demographic information and midwifery records questionnaire. The DASS‐21 and the researcher‐made questionnaire will be completed before and 8 to 10 weeks after the intervention by two groups.

The demographic information and midwifery records questionnaire has two sections: demographic‐social information and specifications of AASTs. The demographic‐social information section includes questions about age, education level, employment, economic status, year of marriage and the number of children and will be completed through face‐to‐face interviews upon entering the study. The specifications AASTs section, which will be completed 8–10 weeks after the intervention, includes questions about the timing and results of the AASTs.

The Depression, Anxiety and Stress Scale (DASS‐21) will be used to measure the emotional states of pregnant women. This questionnaire consists of 21 phrases related to the symptoms of negative emotions (depression, anxiety and stress). Each question is scored from zero (does not apply to me at all) to 3 (applies to me very much). As DASS‐21 is the abbreviated form of the main scale (42 questions), the final score of each of these subscales should be doubled. Questions 21, 17, 16, 13, 10, 5 and 3 are related to the depression subscale; questions 20, 19, 15, 9, 7, 4 and 2 are related to the anxiety subscale; and questions 18, 14, 12, 11, 8, 6 and 1 are related to the stress subscale. The validity and reliability of the DASS questionnaire for all three subscales of depression, anxiety and stress have been confirmed with the scores of 0.91, 0.84 and 0.90, respectively (Lovibond & Lovibond, 1996). The validity and reliability of this questionnaire have also been confirmed in Iran (Sahebi et al., 2005; Samani & Joukar, 2007).

To assess the pregnant women's concerns in the process of undergoing AASTs, a questionnaire will be designed by the researcher using the results of the qualitative part and the literature review. The content validation method will be used to confirm the validity of the designed instrument. For this purpose, content validity will be determined qualitatively and quantitatively. In the qualitative method, the questionnaire will be presented to ten experts and faculty members to be examined in terms of grammar, appropriateness and scoring. In addition, the content validity ratio (CVR) and content validity index (CVI) will be used to evaluate the content validity quantitatively. To determine the reliability of the instrument, the test–retest method will be applied; in this method, the modified questionnaire will be presented to twenty qualified individuals twice with a two‐week interval. The reliability of the instrument will then be measured by calculating the correlation coefficient. Based on the results of the first and second stages of the study, other appropriate tools will be used if necessary.

#### 4.5.10 The implementation method

The quantitative part of the study is a randomized controlled trial to determine the impact of the designed intervention on reducing pregnant women's concerns for undergoing AASTs. After registering the study in the Iranian Registry of Clinical Trials (IRCT), obtaining the necessary approval from the School of Nursing‐Midwifery and the Ethics Committee and presenting the recommendation letter to hospitals and health centres, affiliated with Tehran University of Medical Sciences, sampling will start at these centres. Initially, continuous convenience sampling will be implemented, that is, all eligible pregnant women will be enrolled in the study after receiving detailed explanations about the purpose of the study, and signing a written consent form. Next, the samples will be divided into intervention and comparison groups using permuted block randomization. The designed intervention will be presented for the intervention group. The control group will not receive any interventions from the research team during the study. Before and 8–10 weeks after the intervention, the status of the participants in the intervention and control groups will be measured using the questionnaire. Finally, the results before and after the intervention will be analysed using inferential statistical methods. A telephone follow‐up will be performed to ensure that all intervention participants receive the intervention and also to ensure the completion of questionnaires in both groups. The participants' data will be stored on a computer and kept confidential. Each participant will have a unique code to avoid double data entry. Participants will be assured of the confidentiality of information.

#### 4.5.11 Data analysis

After completing the questionnaires, statistical analysis will be performed using SPSS16.0 on a Windows system (IBM SPSS V.16.0.0). Continuous variables will be presented in the form of mean ±standard deviation (*SD*), and categorical variables will be reported in the form of frequency (percentage). The normality of the variables will be checked using the Kolmogorov–Smirnov test. The chi‐square test or Fisher's exact test, independent sample *t* test and paired *t* test will be used to check the homogeneity of qualitative variables, compare quantitative variables between groups and compare the pre‐/posteffect in each group, respectively.

### 4.6 Strategies to implement the legitimation criteria

The term ‘legitimation’ was coined by Onwuegbuzie and Johnson to assess mixed‐methods validity and was revised the existing criteria in subsequent publications (Collins et al., 2012; Onwuegbuzie et al., 2011; Younas et al., 2020). In the present mixed‐methods study, this approach will be used for implementing the legitimation criteria, considering the following strategies:

#### 4.6.1 Inside–outside legitimation

The content analysis will be used to precisely explain the emic perspective of pregnant women in AASP. To ensure concordance and reduce bias regarding the participants' etic and emic perspectives, two researchers will separately work on the themes and categories. To provide a balanced explanation of the emic and etic perspectives, the themes and categories from the qualitative phase, the results of the quantitative phase and the researchers' mixed‐method interpretations will be presented in joint displays.

#### 4.6.2 Paradigmatic/philosophical legitimation

James' pragmatism was used as research guidance because it fits qualitatively driven mixed‐methods. Before the conduction of the study, the pragmatic outcomes of the methods were determined, and the following questions were presented:

•Should we use an existing intervention for the study or design a new one?
•Should we use content or thematic analysis?

Finally, it was decided to design a new intervention and use content analysis to explain pregnant women's perspectives.

#### 4.6.3 Commensurability approximation legitimation

We cooperated with researchers who were experienced in qualitative, quantitative and mixed‐method studies to make it easier to move between these approaches. These qualitative and quantitative research experts will be supposed to explain the emic and etic views, respectively. The mixed‐methods researchers will help us to select the best methodology.

#### 4.6.4 Weakness minimization legitimation

The literature review showed a gap in qualitative studies and interventions about pregnant women's concerns in AASP. We concluded that a combination of methods with an initial qualitative stage is necessary to study the challenges. The qualitative approach gives us accurate information about pregnant women's experiences in AASP. The obtained themes and categories can be used to develop an appropriate intervention for this context. In this way, experts will prioritize the themes and categories and evaluate the designed intervention based on content validity.

#### 4.6.5 Sequential legitimation

Due to the lack of a qualitative study about pregnant women's concerns in AASP and also to achieve a good understanding of pregnant women's experiences, the first part of the study will be done qualitatively. The higher importance of the qualitative phase is because it builds on the quantitative phases.

#### 4.6.6 Conversion legitimation

Themes, categories and pregnant women's perspectives will be used to design an intervention. The themes will be compiled and prioritized with the help of experts to design the intervention accordingly.

#### 4.6.7 Sample integration legitimation

The qualitative and quantitative stages of the study will be conducted by different groups of participants. To achieve data saturation, purposive sampling and an appropriate sample size will be used in the qualitative part. In addition, large sample size will be used in the quantitative part. It will be ensured that the samples fit the target population.

#### 4.6.8 Pragmatic legitimation

Finally, the following poststudy questions will be presented:

•Did the study solve any practical problems?
•Did the study answer the research questions?

### 4.7 Integration of the qualitative and quantitative data

The results of the qualitative and quantitative phases of the study will be integrated. Finally, the researcher will interpret to what extent quantitative results have been able to expand or develop the initial qualitative findings.

## 5 DISCUSSION

According to the guidelines of the American College of Obstetrics and Gynecology, screening for aneuploidy should be the patient's informed choice while providing help for her to make a decision based on clinical conditions, values, interests and goals. The purpose of a screening test is to provide information, not to dictate a process (ACOG & May, 2016).

Screening tests are routinely requested for all pregnant women in Iran. If a midwife or gynaecologist does not request a screening test and the baby is born with Down syndrome, there will be legal ramifications (Abbasi et al., 2012). Given the limited time required for legal abortion in Iran (until 18 WK and 6 days of gestational age), these tests are requested promptly with no time devoted to training. Due to the high cost of the tests, individuals refuse to undergo the tests if they are not provided with proper information.

A screening test is a method that can identify seemingly healthy people who are at high risk for a disease or disorder, yet there is no guarantee that it will not occur. The majority of high‐risk women receive normal diagnostic results. Therefore, the unreasonable concern is the main psychological and social cost that these women pay for screening tests. Consequently, exceptional attention should be paid to concerns associated with AASTs. Promoting professional support during these phases might help control and manage these concerns and associated distress. Therefore, the researcher intends to design an appropriate and comprehensive intervention to provide the necessary conditions for mothers to make informed decisions and reduce their concerns in the AASP. As women's health concerns are culture‐ and context‐based and cannot be assessed by quantitative research alone, the research team will conduct a multi‐phased study. First, we will examine women's concerns about AASTs using a qualitative approach. Afterwards, using the experiences of these women and the experts' opinions, we will offer an intervention to reduce their concerns in one of the most critical stages of their lives. The results of this study can serve as a basis for policy‐making and interventional strategies in healthcare systems to alleviate pregnant women's concerns and improve their mental health by providing knowledge about their concerns in AASTs.

## 6 LIMITATIONS

There are two limitations to this study. In the qualitative phase, the women might be reluctant to participate in the research because of the sensitivity of the subject or the undesirable spiritual condition following high‐risk test results. Regarding such cases, the researcher will attempt to gain the participants' trust and confidence and finally eliminate this limitation by explaining the significance of the research, establishing a friendly relationship with participants, spending enough time in practice, and considering ethical principles. Furthermore, in the quantitative phase, there will be the likelihood of a lengthy sampling time due to a reduction in the number of pregnant women referring to medical centres because of fear of becoming infected with COVID‐19. To reduce this limitation, if the pregnant women have access to the Internet, the completed questionnaires will be obtained through cyberspace or email; besides, for those who do not have access to the Internet, the researcher will be completed the questionnaires by phone.

## CONFILICT OF INTEREST

The authors announce that they have no conflict of interest.

## ETHIC APPROVAL

This protocol has been approved by the Research Ethics Committee of the Tehran University of Medical Sciences, Tehran, Iran (date: 2018–09–01, approval ID: IR.TUMS.FNM.REC.1397.099). Written informed consent will be obtained from each participant.

## Data Availability

Not applicable.
